# A Randomized Comparison of Clothing Removal Techniques in a Simulated Trauma Patient Exposure

**DOI:** 10.7759/cureus.29237

**Published:** 2022-09-16

**Authors:** Caleb Cummings, Aaron Sibley, Trevor Jain, Brent Nicholson

**Affiliations:** 1 Paramedicine, University of Prince Edward Island, Charlottetown, CAN; 2 Emergency Medicine, Dalhousie University/University of Prince Edward Island, Charlottetown, CAN; 3 Paramedicine, Holland College, Charlottetown, CAN

**Keywords:** trauma shears, cutting hook, ems, simulation, clothing removal, paramedic, emergency medical service, major trauma

## Abstract

Introduction

Trauma shears are commonly used by emergency medical services (EMS) providers to remove clothing from patients and expose underlying traumatic injuries. Other tools exist that may be more effective, but they are largely untested. This study compared the use of trauma shears versus two cutting hooks in removing clothing from a simulated trauma patient.

Methods

We recruited 18 paramedic students to participate in a cross-over study designed to remove clothing from a wholly dressed full-body training mannequin using trauma shears (with the cut-and-rip (CAR) technique) and two cutting hooks (S-Cut QE (ES Equipment AB, Nol, Sweden) and the Talon Rescue Emergency Clothing Knife (TRECK+, Talon Rescue, Farmington, CT, USA)). We determined the order of the tools using a three-by-three Latin square and randomized participants equally between possible orders to minimize carryover effects. We recorded times for total clothing removal and the removal of clothing from the upper and lower body, respectively. We employed a mixed-effects analysis of variance (ANOVA) to determine any differences between tools.

Results

Removal time was significantly faster with the S-Cut QE compared to the CAR technique and TRECK+ (mean 78 seconds, 95% confidence interval (CI) 52-103 vs. 142 seconds, 95% CI 117-167, vs. 209 seconds, 95% CI 184-235, p<0.001). The S-Cut QE was significantly faster than the CAR technique and TRECK+ for upper body clothing removal (mean 47 seconds, 95% CI 30-64 vs. 92 seconds, 95% CI 75-109, vs. 131 seconds, 95% CI 115-148, p<0.001) and the S-Cut QE and CAR were significantly faster than TRECK+ for lower body clothing removal (mean 25 seconds, 95% CI 11-38 and 44 seconds, 95% CI 31-58 vs. 71 seconds, 95% CI 58-85, p<0.001). Most (78%) participants preferred the S-Cut QE over other tools.

Conclusion

The S-Cut QE removed clothing from a simulated trauma patient faster than both the CAR and TRECK+. Emergency medical services (EMS) agencies should consider adding a cutting hook to their standard trauma kit.

## Introduction

Trauma is a leading cause of death in Canada, particularly among children and young adults [1]. This is also true in the United States where unintentional injuries were the fourth most common cause of death in 2020 [2]. When dealing with traumatic injuries, clinicians, including paramedics, are taught to remove the patient's clothing to rapidly identify and manage life threats [3]. Using the most efficient method to remove clothing may be critical for injuries that require time-sensitive interventions, for example, decompression of a tension pneumothorax or the application of a tourniquet in massive peripheral hemorrhage. Hemorrhage, including injuries to the extremities, has been identified as a major cause of potentially preventable prehospital trauma deaths [4]. Even small reductions in the time to expose a trauma patient might be important in scenarios where multiple patient assessments are required, such as multiple or mass casualty incidents, or when provider safety is an issue including care under fire [5]. In addition to trauma, rapid exposure to the chest is essential in cardiac arrest to prevent delays in defibrillation [6]. Unfortunately, minimal evidence exists to suggest the best technique and/or tool for clothing removal.

Trauma shears are blunt-tipped scissors capable of cutting through various materials and for decades they have been a standard piece of equipment in prehospital trauma kits. A single universally recommended technique for the use of trauma shears does not exist; however, the cut-and-rip (CAR) method has previously been demonstrated to be faster than a standard cutting technique in removing clothing from a simulated trauma patient [7]. Cutting hooks, in comparison, are relatively new pieces of equipment that are purported by manufacturers to be more efficient than trauma shears but are generally more costly and not supplied as standard equipment by many prehospital services. These are knives with a blade inserted into a curved portion allowing the user to cut one or more layers of clothing by pulling the knife towards themselves. Previous studies found that a cutting hook may perform better than trauma shears when removing a single layer of clothing from a simulated trauma or cardiac arrest patient [8-10].

This article was previously presented as an abstract as part of Dalhousie University's Emergency Medicine Services (EMS) Research Day on October 27, 2020, and as a poster for the Trauma Association of Canada's conference from April 12 to 16, 2021.

## Materials and methods

Study design/ethics

This study was a randomized, cross-over trial, comparing two cutting hooks to the CAR technique in removal of clothing from a simulated trauma patient. The Holland College Applied Research Ethics Board approved this study and all participants provided informed written consent.

Participants/setting

We recruited a convenience sample of paramedicine students from the Holland College Paramedicine Diploma Programs. Students were enrolled in either in the Primary Care Paramedic (PCP) or Advanced Care Paramedic (ACP) program. The PCP program at Holland College is a two-year diploma combining both didactic and practical experiences that focus on the foundational knowledge and basic life support skills necessary to care for patients in the prehospital environment. The ACP program requires an additional year of training, and it is designed to build upon critical thinking and teach advanced life support skills. We collected participant demographics, including age, gender, and years of practice (if applicable). All trials took place in the paramedicine program labs at Holland College in Charlottetown, Prince Edward Island, Canada.

Interventions

**Figure 1:**
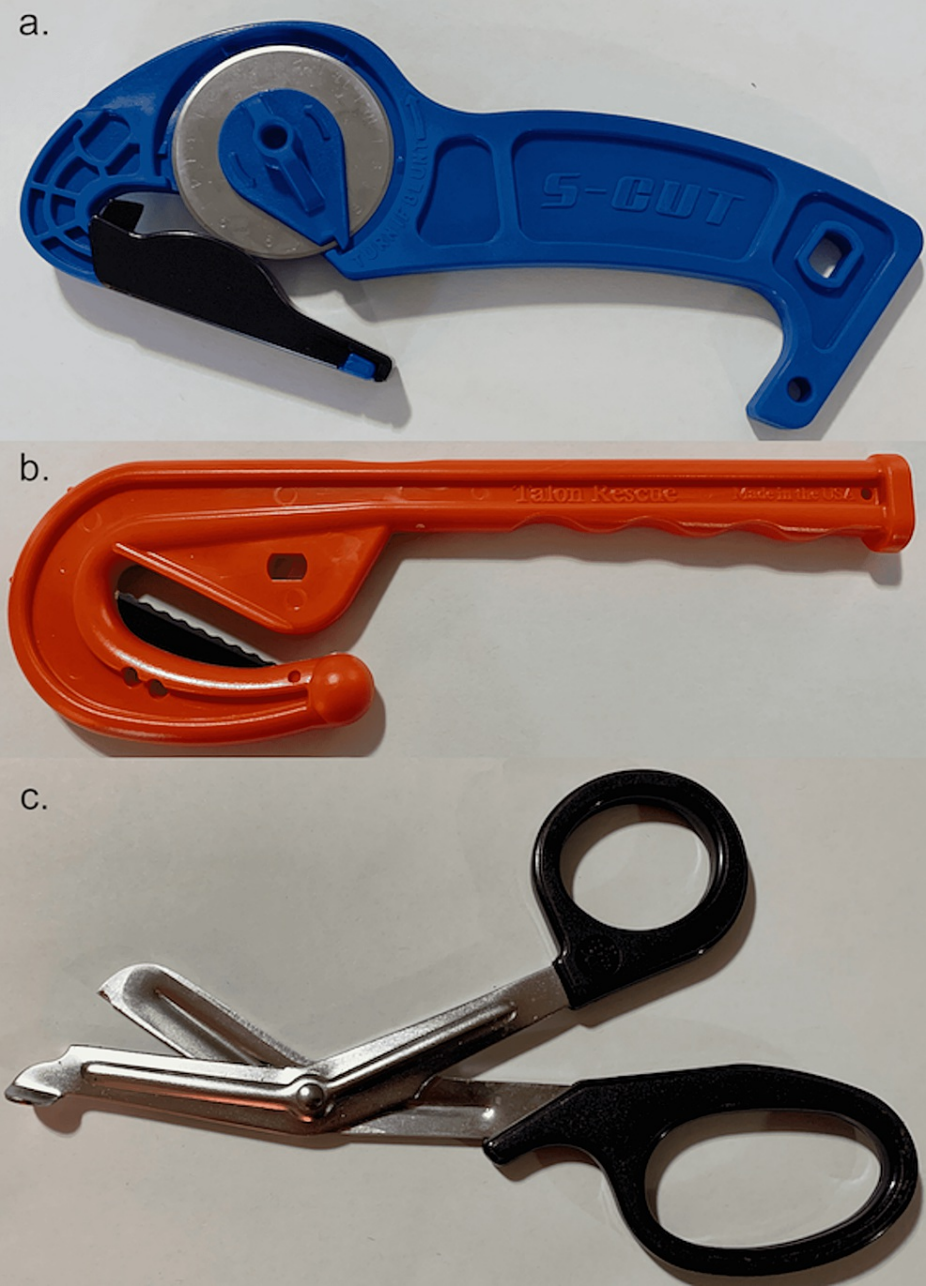
Cutting tools a: The S-Cut QE, b: The Talon Rescue Emergency Clothing Knife (TRECK+), c: Trauma shears

We selected the CAR technique for evaluation because of the universal use of trauma shears in prehospital medicine and our prior research showing it to be superior to a simple cutting technique [7]. In order to select comparators, we completed an online search for commercially available cutting hooks. We selected two different hooks (Figure 1) based on price point (we considered less than $10/unit to be a low price point and greater than $50/unit to be a high price point) in addition to the availability of educational material and evidence to support the use of the hook. The S-Cut QE (ES Equipment AB, Nol, Sweden) has previously been shown to be faster than trauma shears in removing clothing from a simulated patient [10], and it is at the upper range of cost for the cutting hooks considered for the study. The Talon Rescue Emergency Clothing Knife (TRECK+, Talon Rescue, Farmington, CT, USA) provided online video demonstrations of use and it is one of the least expensive tools we found available; however, it has not been evaluated in a published research study. Neither company was aware of the study and the tools were obtained at the full purchase price. We provided all participants with a one-hour information session on the purpose of the study as well as the requirements for participation. Using video demonstrations based on the published CAR technique and manufacturer recommendations for each cutting hook, we instructed students on proper clothing removal [7]. We also gave participants the opportunity to practice with each technique/tool using a piece of clothing. No practice occurred on the mannequin.

**Figure 2:**
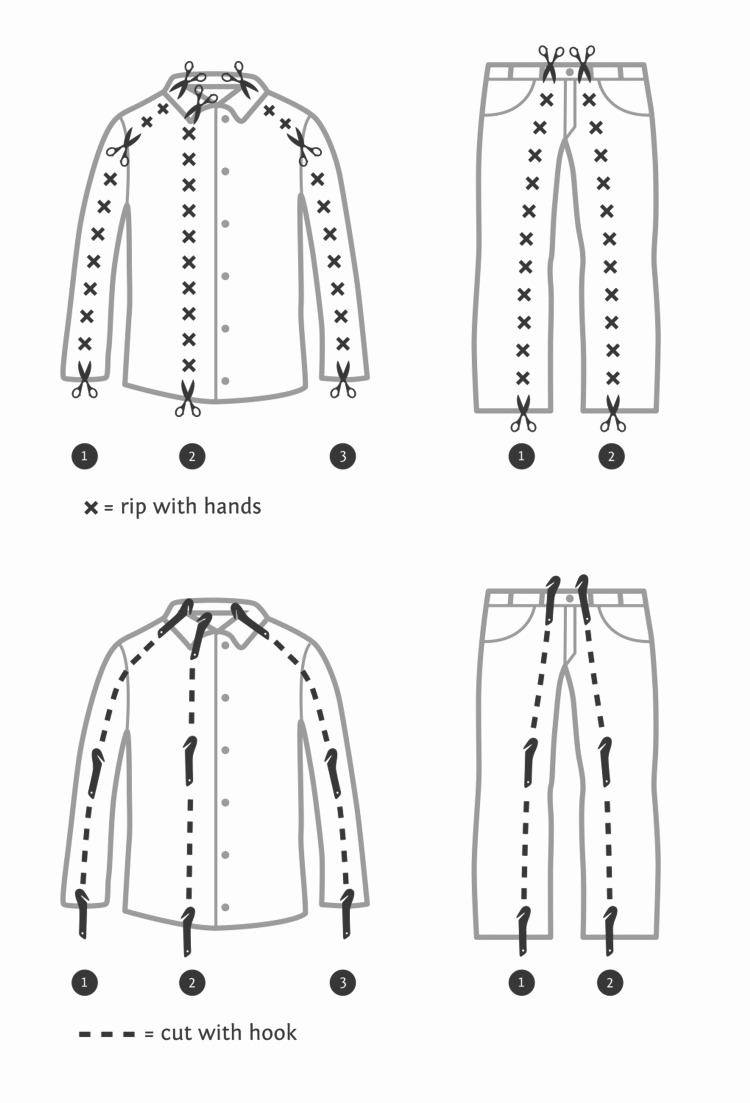
Standardized procedures for clothing removal with trauma shears and cutting hooks Adapted from Figure 1 of Sibley et al. [7}. Used with permission from the authors and the Canadian Journal of Emergency Medicine.

Participants were assigned a number from one to 18 in a random order determined by an online number randomizer. To begin, participants handed their data collection sheet to a trained volunteer adjudicator/timer who matched their randomized number to a corresponding tool order. Participants were blinded to the tool order before entering the room and were blinded to other participants’ attempts. Adjudicators were blinded to the tool order prior to receiving the participant’s randomized number. The participant then completed three trials back-to-back, one with each technique/tool. We used an adult full-body training mannequin (MegaCode Kelly, Laerdal Medical, Stavanger, Norway) placed supine on an ambulance stretcher (Ferno Proflex, Ferno-Washington Inc., Wilmington, OH, USA) as the simulated trauma patient. Clothing consisted of a pair of jeans, a short-sleeve t-shirt, and a long sleeve collared button-up. Jeans ranged in waist size from 30 to 36 inches and an inseam of 30 to 34 inches; shirts were size medium and large. Sizes were distributed evenly among the three groups. Clothing removal for all methods proceeded in an organized fashion, starting with the upper body and followed by the lower body. With cutting hooks, cuts were made along both sleeves and down the middle of the shirts. For the CAR, participants used the previously described technique but used their hands to rip the fabric after the initial cut (Figure 2). We did not allow participants to rip buttons on the long sleeve shirt, but they could cut multiple layers at once. Using the cutting hook for the lower body, jeans were removed with a single cut from the waist down each leg, avoiding the pockets. The CAR followed the same path but went from an initial cut at the bottom of the leg and ripped up to the waist where a final cut was performed (Figure 2). The timer used the stopwatch function on an iPhone 6s (Apple Inc., Cupertino, CA, USA) to record the times. Timing started when the blade touched clothing and stopped when both arms, the entire chest, both legs, and the pelvis were entirely visible, and the participant had rolled the mannequin to check the back. Each of the purchased tools was used in nine separate trials. We rotated the S-Cut blade every two trials to expose a new cutting edge.

Upon completion of all three trials, we asked participants to complete a short survey. Participants ranked the tools in order from one to three with one being the easiest to use and three being the most difficult. The participants then described which tool they would prefer to use in the field and why.

Outcome measures

The primary outcome was the time (in seconds) to complete exposure of the simulated patient including a back check. Secondary outcomes included time to remove clothing from the upper body and lower body, respectively, plus a ranking of the tools by the participants.

Sample size and randomization

We estimated that using a cross-over design, 13 participants would provide 80% power to detect a clinically significant difference of 30 seconds between the two techniques (alpha=0.05). The clinically significant difference was previously determined by Sibley et al [7]. In order to account for potential carryover effects related to tool order, we employed a three-by-three Latin square, thus requiring a total of 18 participants. We randomly allocated participants to a specific tool order using an online random number generator.

Statistical analysis

We analyzed all three outcomes separately using a mixed-effects analysis of variance (ANOVA) with participants as a random effect and the tool and trial number as fixed effects. We evaluated model assumptions by residual plots and found they were met to a satisfactory degree. Pairwise comparisons were conducted by the Tukey method. All analyses were conducted using Minitab 19 (Minitab Inc., State College, PA, USA).

## Results

We recruited 18 participants i.e., 13 ACP students (72%), and five PCP students (28%) that completed all three trials. The ACP students had a mean of three years of paramedic practice while the PCP students only had experience from educational exposures. The mean participant age was 26 years (Table 1).

**Table 1:** Characteristics of the 18 paramedic students ACP: Advanced care paramedic *Clinical experience as a paramedic only recorded for ACP students

Characteristic	N (%)
Age (mean years)	26
ACP	13 (72)
Clinical experience* (mean years)	3

For the primary outcome of time to complete clothing removal (Table 2), the S-Cut QE was the fastest with a mean difference of 64 seconds compared to the second fastest, the CAR technique (mean 78 seconds, 95% CI 52-103 vs. 142 seconds, 95% CI 117-167, p<0.001). The TRECK+ was the slowest, with a mean difference of 67 seconds compared to the CAR technique (mean 209 seconds, 95% CI 184-235 vs.142 seconds, 95% CI 117-167, p<0.001).

**Table 2:** Mean times to clothing removal for individual techniques *Total time includes rolling the mannequin for a back check CAR: Cut-and-rip, TRECK+: Talon Rescue Emergency Clothing Knife, CI: Confidence interval

	Time in Seconds (95% CI)
S-Cut QE (n=18)	
Total*	78 (52-103)
Upper body	47 (30-64)
Lower body	25 (11-38)
CAR (n=18)	
Total*	142 (117-167)
Upper body	92 (75-109)
Lower body	44 (31-58)
TRECK+ (n=18)	
Total*	209 (184-235)
Upper body	131 (115-148)
Lower body	71 (58-85)

For the secondary outcome, removal of clothing from the upper body, there was a statistically significant mean difference of 45 seconds between the S-Cut QE and the CAR technique (mean 47 seconds, 95% CI 30-64 vs. 92 seconds, 95% CI 75-109, p<0.001) and the S-Cut QE and the TRECK+ (mean 47 seconds, 95% CI 30-64 vs. 131 seconds, 95% CI 115-148, p<0.001). The difference between the CAR and the TRECK+ was also statistically significant (mean 92 seconds, 95% CI 75-109 vs. 131 seconds, 95% CI 115-148, p<0.001).

For removal of clothing from the lower body, the S-Cut QE was faster than the CAR technique with a difference of 19 seconds (mean 25 seconds, 95% CI 11-38 vs. 44 seconds, 95% CI 31-58, p=0.106); however, this difference was not statistically significant. The TRECK+ (mean 71 seconds, 95% CI 58-85) was significantly slower than both the S-Cut QE (p<0.001) and CAR technique (p<0.001).

Despite never having used the tool, most of the participants (78%) preferred the S-Cut QE, citing the ease of use and cutting ability. All other participants (22%) preferred the trauma shears with CAR. These participants commented on their familiarity with the tool as well as the versatility, reliability, and ease of use of shears.

## Discussion

Our study demonstrated that the S-Cut QE was faster than both the CAR technique and TRECK+ in the removal of clothing from a simulated trauma patient. The S-Cut QE was more than one minute faster than the CAR in time to total body exposure. These results indicate that the use of a cutting hook can potentially decrease the time to locate critical injuries. Moreover, this effect may be additive in the case of poly-trauma or multiple/mass casualty incidents where these actions are performed multiple times.

While our results support previous studies demonstrating that cutting hooks exposed simulated trauma patients more rapidly than trauma shears, our times to exposure were generally slower compared to other studies [8-10]. The reasons for these differences in times are likely multi-factorial. We used multiple layers of clothing for the upper body compared to other cutting hook studies [8,9]. Our participants were only shown video directions on how to use the tools and were not able to practice on the mannequin before participating in a trial. Both the TRECK+ and CAR require a specific technique to use effectively, and minimal practice may have prevented the development of a refined technique. This may be important in areas where major traumas are not common as skill retention and degradation have been identified as issues with other prehospital interventions [11]. The procurement of a tool that can be used intuitively may provide the greatest benefit as minimal training is required for skill upkeep.

The S-Cut group contained two outliers. In both instances a sharp pull on the cutting hook caused the round blade on the S-Cut to roll back over a dull section of the blade, preventing the clothing from cutting and causing a jam that was difficult for the participant to resolve. How often this is likely to happen in a larger sample is uncertain. The other tools were reliable but provided slower times in our study. There may be a trade-off between cost, reliability, and ease of use in these tools that practitioners will need to consider when choosing a tool.

Strengths/limitations

The key strengths of our study include a randomized cross-over design to compare the use of techniques by the same operator and the comparison of a previously tested trauma shears method with multiple cutting hooks. We were unable to blind participants and evaluators to the intent of the study or specific tool, although, both participants and evaluators were unaware of the order in which they would use the tools prior to the trial. Participants only had a short time to practice with the tools; developing proper technique and comfort with the tools may take additional experience and practice beyond that included in our study. This study is limited by the application of techniques in a simulated setting. We did not consider potential differences in live patient body habitus, positioning, or the impact of scene hazards. Finally, although there are several cutting tools available commercially, we were only able to test two. Further study is required to compare a broader number of available tools.

## Conclusions

This study showed that paramedic students using the S-Cut QE cutting hook were faster at exposing a simulated trauma patient when compared to the CAR technique with standard trauma shears and the TRECK+ cutting hook. Cutting hooks have the potential to improve the time to remove clothing from injured patients and emergency services should strongly consider implementing a cutting hook as a part of their trauma kits. However, emergency services must balance cost, reliability, likely applications, and the need for training to select a cutting hook that fits the requirements of their service.
